# Integrating early clinical exposure, digital simulation, and soft-skill development into undergraduate paediatric dentistry curricula

**DOI:** 10.3389/froh.2026.1794385

**Published:** 2026-03-06

**Authors:** Abdul Rauf Badrul Hisham

**Affiliations:** 1 Centre of Paediatric Dentistry and Orthodontics Studies, Faculty of Dentistry, Universiti Teknologi MARA (UiTM), Sungai Buloh Campus, Jalan Hospital, Sungai Buloh, Selangor, Malaysia; 2 Kulliyyah of Dentistry, International Islamic University Malaysia, Kuantan, Pahang, Malaysia

**Keywords:** curricula, curriculum development, dental education, digital simulation 8, paediatric dentistry

Current emerging evidence necessitates a comprehensive overhaul of the undergraduate paediatric dentistry curriculum. Recent studies demonstrate that the early inclusion of paediatric content strengthens both technical competence and the appreciation of the specific needs of child patients (1). Yet, a scoping review of dental education has identified persistent gaps, including a poor correlation between basic science and clinical phases, insufficient patient-care experience, and limited exposure to geriatric, specialised paediatric, and medically compromised cases (2). These deficits are significant contributors to the anxiety graduates face when confronting independent practice (2).

To bridge these gaps, curricula must expand community-based rotations and introduce elective modules targeting specialised areas such as interceptive orthodontics, sleep medicine, and practice management (2). Furthermore, instructional design that incorporates digital simulations and case-based exercises—particularly for primary tooth identification—has been proven to boost student engagement and the retention of crucial knowledge (1, 2). The rapid adoption of telehealth platforms further enables students to observe remote consultations, effectively preparing them for an increasingly digitised paediatric practice environment (1).

Assessment of these new learning modalities requires robust standard-setting methods. The Modified Angoff approach, incorporating a reality-check component, currently offers the highest reliability for defining competency-based cut scores in health-professional assessments (3).

Equally critical is the development of soft skills. Evidence indicates that communication, empathy, critical thinking, and interprofessional teamwork are indispensable for delivering patient-centred paediatric care (4). Embedding structured soft-skill training early in the curriculum, and reinforcing it through interdisciplinary collaborations with nutrition, mental health, and public health professionals, will produce graduates who are both clinically proficient and compassionate.

In summary, the future of undergraduate paediatric dentistry hinges on a curriculum that (i) introduces paediatric concepts early, (ii) provides ample and diverse clinical exposure, (iii) leverages modern instructional technologies, (iv) applies rigorous, evidence-based assessment standards, and (v) cultivates essential soft skills. **I urge** educators and policymakers to adopt these evidence-based recommendations to ensure that tomorrow's dentists are fully prepared to improve oral health outcomes for children.
